# Stub1 promotes degradation of the activated Diaph3: A negative feedback regulatory mechanism of the actin nucleator

**DOI:** 10.1016/j.jbc.2024.107813

**Published:** 2024-09-23

**Authors:** Cui Qiu, Linqing Zhang, Chenxuan Yong, Ruixing Hu, Yuecen Sun, Busong Wang, Lei Fang, Guang-Jie Zhu, Qing Lu, Junguo Wang, Xiaofeng Ma, Luping Zhang, Guoqiang Wan

**Affiliations:** 1 MOE Key Laboratory of Model Animal for Disease Study, Department of Otolaryngology Head and Neck Surgery, Jiangsu Provincial Key Medical Discipline (Laboratory), The Affiliated Drum Tower Hospital of Medical School, Model Animal Research Center of Medical School, Nanjing University, Nanjing, China; 2 Bio-X Institutes, Key Laboratory for the Genetics of Developmental and Neuropsychiatric Disorders (Ministry of Education), Shanghai Jiao Tong University, Shanghai, China; 3 State Key Laboratory of Pharmaceutical Biotechnology, Jiangsu Key Laboratory of Molecular Medicine, National Resource Center for Mutant Mice of China, Medical School, Nanjing University, Nanjing, China; 4 Research Institute of Otolaryngology, Nanjing, China; 5 Department of Otolaryngology-Head and Neck Surgery, Affiliated Hospital, Nantong University, Nantong, China

**Keywords:** Diaph3, Stub1, actin polymerization, autoinhibition, protein stability, negative feedback, hearing loss

## Abstract

The formin protein Diaph3 is an actin nucleator that regulates numerous cytoskeleton-dependent cellular processes through the activation of actin polymerization. Expression and activity of Diaph3 is tightly regulated: lack of Diaph3 results in developmental defects and embryonic lethality in mice, while overexpression of Diaph3 causes auditory neuropathy. It is known that Diaph3 homophilic interactions include the intramolecular interaction of its Dia-inhibitory domain (DID)-diaphanous autoregulatory domain (DAD) domains and the intermolecular interactions of DD-DD domains or FH2-FH2 domains. However, the physiological significance of these interactions in Diaph3 protein stability and activity is not fully understood. In this study, we show that FH2-FH2 interaction promotes Diaph3 activity, while DID-DAD and DD-DD interactions inhibit Diaph3 activity through distinct mechanisms. DID-DAD interaction is responsible for the autoinhibition of Diaph3 protein, which is disrupted by binding of Rho GTPases. Interestingly, we find that DID-DAD interaction stabilizes the expression of each DID or DAD domain against proteasomal-mediated degradation. Disruption of DID-DAD interaction by RhoA binding or M1041A mutation causes increased Diaph3 activity and accelerated degradation of the activated Diaph3 protein. Further, the activated Diaph3 is ubiquitinated at K1142/1143/1144 lysine residues by the E3 ligase Stub1. Expression of Stub1 is causally related to the stability and activity of Diaph3. Knockdown of Stub1 in mouse cochlea results in hair cell stereocilia defects, neuronal degeneration, and hearing loss, resembling the phenotypes of mice overexpressing Diaph3. Thus, our study reports a novel regulatory mechanism of Diaph3 protein expression and activity whereby the active but not inactive Diaph3 is readily degraded to prevent excessive actin polymerization.

Formin homology proteins (formins) are a highly conserved family of cytoskeletal regulatory proteins. Formin activity is required *in vivo* for a diverse array of cellular functions such as stress fiber formation, cytokinetic ring formation, cell-cell junction assembly, induction of cell polarity, and activation of the MAL/serum response factor (SRF) signaling pathway (1, 2, 3, 4, 5). At the core of all these activities is the ability of formins to regulate actin cytoskeletal dynamics. Formins can be subdivided into families based on their associated regulatory domains. The diaphanous-related formins (DRFs) are distinguished by the presence of two interacting regulatory domains, an N-terminal GTPase binding domain and a C-terminal diaphanous autoregulatory domain (DAD) (6, 7). One of the best characterized DRFs is the mammalian homolog of diaphanous, Diaph3.

Auditory neuropathy is a rare form of deafness characterized by a missing or abnormal auditory brainstem response, but retaining the function of outer hair cells. Autosomal dominant nonsyndromic auditory neuropathy (AUNA1) was caused by a mutation in the 5′UTR region of the *DIAPH3*. The c-172g > A mutation in the 5′UTR region of *DIAPH3* resulted in a significant (approximately 1.5-fold) increase in protein expression; in addition, c-172g > A mutation was sufficient to drive the overexpression of luciferase reporter genes (8, 9). Transgenic mice that overexpressed Diaph3 displayed altered inner hair cell stereocilia and reduced auditory brainstem responses, resembling the human auditory neuropathy phenotype (10). These findings suggest that DIAPH3 overexpression or overactivation results in auditory neuropathy associated with abnormalities in hair cell stereocilia. In contrast, mice deficient in Diaph3 resulted in developmental defects but apparently did not affect hearing acuity (11). Thus, the expression and activity of Diaph3 is tightly and precisely regulated in physiological states, as either Diaph3 overexpression (hyperactivity) or deficiency (hypoactivity) may exhibit pathological consequences.

The FH1 and FH2 (FH12) domains of Diaph3 promote actin polymerization, where the FH1 domain first binds to profilin which recruits G-actin, and then the dimeric FH2-FH2 domain formed by Diaph3 intermolecular interaction will catalyze the polymerization of G-actin to F-actin (12). Due to the pathological consequences of altered Diaph3 activities, there are several intracellular inhibitory pathways to contain aberrant Diaph3 activity. In Diaph3, the DAD domain of C terminus and Dia-inhibitory domain (DID) domain of N terminus interact to form intramolecular interaction (autoinhibition), a process regulated by Rho GTPase (13, 14). After the formation of autoinhibitory interaction, the FH12 domain of C terminus is blocked and the actin polymerization activity is inhibited. In addition, the DD-DD domains of N terminus form intermolecular interaction, which may also inhibit its actin polymerization activity (13, 15). W630A mutation had been reported to abolish intermolecular interaction of the FH2-FH2 domain, resulting in loss of Diaph3 function (16). In contrast, the M1041A mutation abolishes DID-DAD autoinhibition, causing uncontrolled overactivation of Diaph3 (17). As these intramolecular and intermolecular interactions are critical for the regulation of Diaph3 activity, identifying mechanisms and consequences for spatiotemporal regulations of these intramolecular and intermolecular interactions may present novel therapeutic targets for treatment of deafness caused by Diaph3 mutations.

In this study, we found that FH12-FH12 interaction promoted Diaph3 activity, while DID-DAD and DD-DD interactions inhibited Diaph3 activity through distinct mechanisms. Importantly, our study reveals a novel negative feedback regulatory mechanism of Diaph3 protein stability and activity whereby the active but not autoinhibited (inactive) Diaph3 is readily ubiquitinated by the E3 ubiquitin ligase Stub1 and degraded to prevent excessive actin polymerization.

## Results

### Diaph3 activity is regulated by its intramolecular and intermolecular interactions

**Figure 1:**
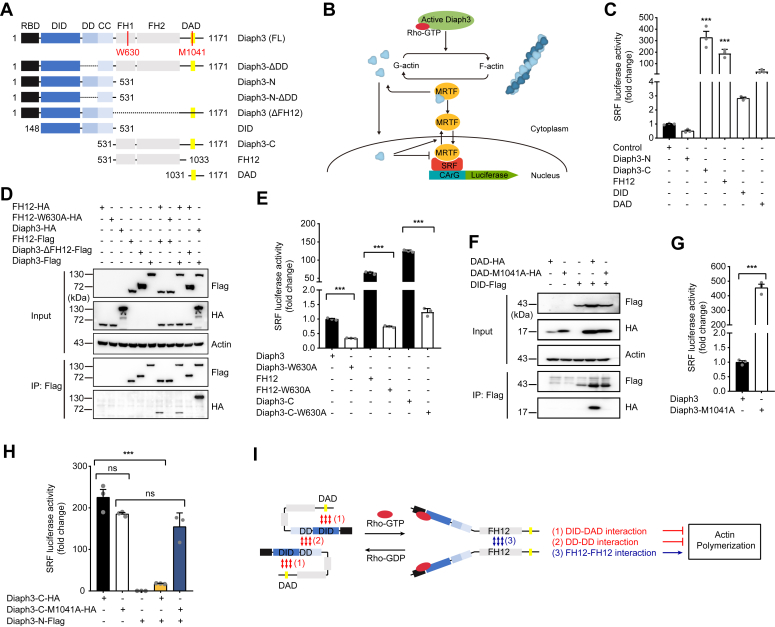
**Diaph3 activity is regulated by its intramolecular and intermolecular interactions.***A*, schematic diagram of Diaph3 domains and constructs. *B*, schematic diagram of actin polymerization activity assayed by the SRF-RE luciferase reporter system. *C*, SRF activity of the truncated Diaph3 constructs transfected in HEK293T cells. *D*, intermolecular interaction of the FH12-FH12 domains was inhibited by the W630A mutation. *E*, intermolecular interaction of the FH12-FH12 domains was required for Diaph3 activity. *F*, intramolecular DID-DAD domain interaction (autoinhibition) was disrupted by the M1041A mutation. *G*, mutation of M1041A in full-length Diaph3 significantly increased SRF activity. *H*, interaction of Diaph3-C and Diaph3-N inhibited the SRF activity of Diaph3-C. *I*, actin polymerization activity of Diaph3 protein induced by binding to the active Rho GTPase is mediated by intermolecular FH12 interactions, but inhibited by both intramolecular DID-DAD interactions and intermolecular DD-DD interactions. Mean ± SEM. ns, not significant, ∗∗∗*p* < 0.001 by unpaired Student’s *t* test. DAD, diaphanous autoregulatory domain; DD, dimerization domain; DID, Dia-inhibitory domain; SRF, serum response factor.

Diaph3 protein is autoinhibited and regulated by the Rho GTPase (12). To study the organization of the Diaph3 regulatory domain in more detail, we constructed Diaph3 plasmids with different truncations and investigated its intramolecular and intermolecular interactions (Fig. 1*A*). Activities of the truncated Diaph3 constructs were first evaluated by a dual luciferase reporting system (SRF-RE luciferase). In the cytoplasm, myocardin-related transcription factor (MRTF; also known as MAL) forms a stable complex with G-actin *via* the RPEL domain, which is present at its N terminus. These regions also include nuclear localization signals. Activation of upstream of MRTF-SRF is regulated by Rho signaling. Stimulation of Rho-GTPases promotes the polymerization of G-actin into F-actin, releases MRTF from G-actin, exposes nuclear localization signals. Free and activated MRTFs translocate to the nucleus, bind to the SRF and promote the transcription of target genes (Fig. 1*B*). This SRF reporter is induced *via* the actin/MAL/SRF pathway in response to changes in actin polymerization dynamics and serves as a rapid and quantitative measure of Diaph3 activity (18, 19). Consist with previous results (20, 21), the reporter assay confirms that the FH1-FH2 (FH12) domains were responsible for the actin polymerization activity (Fig. 1*C*).

We then further evaluated the interactions and consequences of FH12-FH12, DID-DAD, and DD-DD domain interactions on Diaph3 activity. A dimer appeared to be the functional state of formin FH12 domains and was required to promote actin nucleation and elongation (20, 21). We performed coimmunoprecipitation experiments to confirm the FH12-FH12 interaction. As expected, Flag-tagged FH12 was able to coimmunoprecipitate hemagglutinin-tagged WT FH12 but not FH12-W630A (Fig. 1*D*), a mutation reported to disrupt FH12 dimerization (16). FH12-HA interacted with Diaph3-Flag but not Diaph3-ΔFH12-Flag, which indicates that other domains of Diaph3 cannot interact with FH12 (Fig. 1*D*). W630A mutation in a full-length Diaph3, C terminus, and FH12 constructs significantly inhibited Diaph3 activity (Fig. 1*E*), suggesting that intermolecular interaction of the FH12-FH12 domains is critical for Diaph3 activity.

We next examined DID-DAD intramolecular interaction and found that Flag-tagged DID was able to coimmunoprecipitate hemagglutinin-tagged WT DAD but not DAD-M1041A (Fig. 1*F*), a mutation reported to abolish Diaph3 autoinhibition (17) and caused overactivation of Diaph3 (Fig. 1*G*). In addition, the interaction of Diaph3-C (DAD-containing) and Diaph3-N (DID-containing) inhibited the actin polymerization activity of the Diaph3-C, while the M1041A mutation abolished autoinhibition and restored activity of the Diaph3-C (Fig. 1*H*). These results suggested that the intramolecular interaction of DID-DAD (autoinhibition) inhibited the activity of Diaph3, consistent with previous reports (15, 17, 22, 23).

A dimerization domain (DD domain) was sufficient to cause dimerization of N-terminal Diaph3 fragments (15). Lastly, we performed native PAGE experiments to confirm the DD-DD interaction. The results show that the full-length Diaph3 formed an oligomer and the N-terminal domain formed a dimer. However, no dimer was formed after the deletion of DD domain in N terminus, deletion of the DD domain did not affect Diaph3 oligomer formation (Fig. S1*A*). Thus, dimerization of N-terminal Diaph3 fragments was DD domain-dependent. Interestingly, deletion of the DD domain from the full-length Diaph3 also significantly increased its activity, suggesting that intermolecular interaction of the DD-DD domains inhibited the activity of the Diaph3 (Fig. S1*B*). As both interactions of DID-DAD and DD-DD domains inhibited Diaph3 activity, we suspect that deletion of the DD domain may contribute to Diaph3 activity by inhibiting DID-DAD intramolecular interaction (autoinhibition). However, unlike the M1041A mutation, deletion of the DD domain in Diaph3 failed to interact with DAD, suggesting that deletion of the DD domain did not affect intramolecular interaction of the DID-DAD domains (Fig. S1*C*). These results suggested that DD-DD interaction inhibits Diaph3 activity independent of DID-DAD autoinhibition.

Together, we confirm that the intermolecular interactions of FH12 domains are required for the actin polymerization activity of Diaph3, and that the intramolecular interactions of DID-DAD domains and intermolecular interactions of DD-DD domains inhibit Diaph3 activity through distinct mechanisms (Fig. 1*I*).

### DID-DAD interaction-mediated autoinhibition promotes Diaph3 protein stabilization

**Figure 2:**
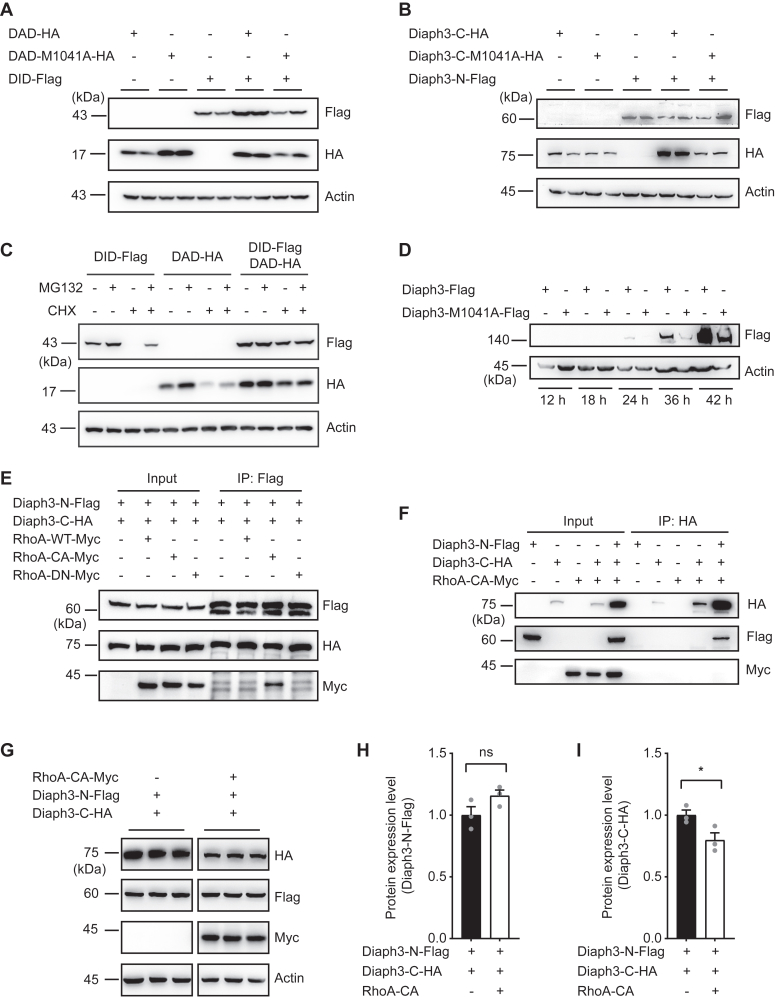
**DID-DAD interaction-mediated autoinhibition promotes Diaph3 protein stabilization.***A*, protein stability of DID and DAD constructs was increased by DID-DAD interaction, and was abolished by M1041A mutation. Western blots of HEK293T cells with DAD-HA, DAD-M1041A-HA, or DID-Flag for Flag, HA and actin. *B*, Diaph3-N stabilized the expression of Diaph3-C but not Diaph3-C-M1041A. *C*, autoinhibition between DID and DAD increased protein stability *via* proteasomal degradation pathways. Cells were treated with 10 μM MG132 (proteasomal inhibitor) and/or 10 μM cycloheximide (CHX) for 4 h. *D*, M1041A mutation promoted degradation of the full-length Diaph3. *E*, RhoA-CA interacted with N-terminal fragment of Diaph3. WT, wildtype; CA, constitutively active; DN, dominant negative. *F*, C-terminal fragment of Diaph3 did not interact with RhoA-CA. Western blots of anti-HA immunoprecipitation of HA-Flag-Myc and whole cell lysate for Flag, HA, Myc from HEK293T cells. *G*, RhoA-CA reduced the stabilization of Diaph3 C-terminal fragment. *H* and *I*, protein quantification of Diaph3-N-Flag and Diaph3-C-HA in panel *G*. Mean ± SD. ns, not significant, ∗*p* < 0.05 by unpaired Student’s *t* test. DAD, diaphanous autoregulatory domain; DID, Dia-inhibitory domain; HA, hemagglutinin.

When DID and DAD domains were coexpressed to study autoinhibition, it is interesting to observe that their protein expression levels increased significantly compared with those expressed alone (Fig. 2*A*). The mutual stabilization of the two domains, however, was diminished by M1041A mutation where DID and DAD-M1041A no longer interacted (Fig. 2*A*). Similarly, coexpression of Diaph3-N promoted stabilization of the Diaph3-C but not Diaph3-C-M1041A (Fig. 2*B*). Effects of the mutual stabilization between DID-DAD appeared to be stronger than that between Diaph3-N and Diaph3-C, indicating that the shorter DID or DAD fragments were less stable in general than the longer N- and C-terminal fragments. Based on these results, we speculate that DID-DAD interaction may facilitate both autoinhibition and stabilization of the Diaph3 protein. To explore whether the increase in protein stability induced by autoinhibition was mediated *via* the proteasomal pathway, we examined the effects of proteasomal inhibitor MG132 and protein synthesis inhibitor cycloheximide (CHX) on DID or DAD expressions. After MG132 treatment, the protein levels of both DID and DAD increased when expressed alone, resembling their mutual stabilization when coexpressed (Fig. 2*C*). In contrast, MG132 treatment did not further increase the protein expressions of DID and DAD when coexpressed, indicating that DID-DAD stabilization was indeed mediated by the proteasomal pathway. The same results were obtained when CHX was cotreated with MG132 (Fig. 2*C*). We further examined the effect of M1041A mutation on the expression of full-length Diaph3. As expected, M1041A mutation resulted in reduced protein levels of Diaph3, compared to the WT Diaph3 beginning at 24 h after transfection (Fig. 2*D*). These results suggested that the intramolecular interaction of DID and DAD in Diaph3 enhanced protein stability *via* the proteasomal pathway.

RhoA GTPases regulate the activity of Diaph3 presumably by competitive binding to the N-terminus of Diaph3 and interruption of the DID-DAD autoinhibition (24). We first predicted the structure of the mouse RhoA-Diaph3 complex using AlphaFold3 (Fig. S2*A*). The model reveals that RhoA interacts with Diaph3 through the GTPase binding region, DID and DAD. Then we compared this predicted RhoA-Diaph3 complex to the crystallographic structure of the RhoC-mDia_N_ complex (Fig. S2*B*). The analysis shows similarities between the structures, particularly in the interface between RhoC and mDia_N_. A sequence alignment of Diaph1, Diaph2, and Diaph3 highlights key residues involved in the interaction (Fig. S2*C*), and most residues (10 of 13) are identical between Diaph1 and Diaph3. The structure and sequence alignment indicate the RhoA-Diaph3 interface is conserved among Rho and Diaphanous families, and that RhoA activates Diaph3 by binding to its N-terminal domains as previously shown (15, 17, 23).

To examine if RhoA binding affects the stability of Diaph3, we coexpressed the Diaph3-N and Diaph3-C with either WT, constitutively active (CA, Q63L) or dominant negative (DN, T19N) RhoA constructs (25). Diaph3-N coimmunoprecipitated with RhoA-CA, but not RhoA-WT or RhoA-DN (Fig. 2*E*); however, Diaph3-C failed to coprecipitate RhoA-CA (Fig. 2*F*). Interestingly, interaction of RhoA-CA with Diaph3-N appeared to reduce the expression of Diaph3-C (Fig. 2, *G*–*I*). Therefore, it is likely that competitive binding of RhoA-CA to the Diaph3-N impaired the stabilization of Diaph3-C when coexpressed.

Together, these results indicated that disruption of DID-DAD interaction (autoinhibition) by M1041A mutation or RhoA binding results in destabilization of the Diaph3 protein, particularly at its C terminus, a potential negative feedback mechanism to restrain the Diaph3 activity.

### E3 ubiquitin ligase Stub1 regulates the activity and degradation of Diaph3-M1041A

**Figure 3:**
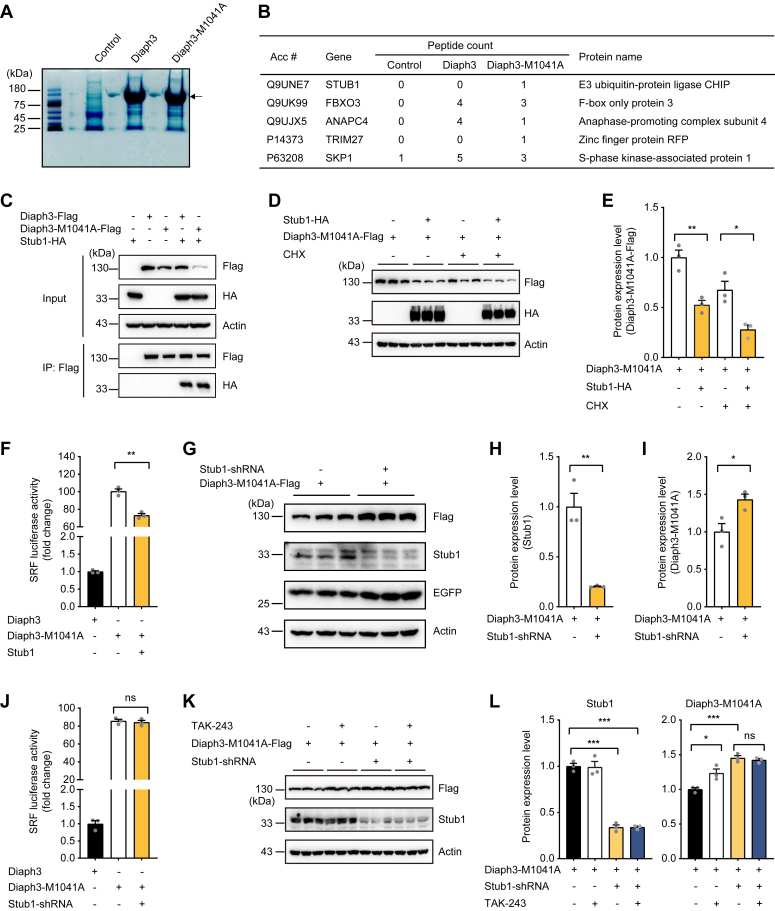
**E3 ubiquitin ligase Stub1 regulates the degradation of Diaph3-M1041A.***A*, coomassie blue gel staining of MG132-treated immunoprecipitated Diaph3 proteins for mass spectrometry. The *arrow* indicates the Diaph3 protein (140 kD). *B*, list of Diaph3-interacting E3 ubiquitin ligases from mass spectrometry data. *C*, WT Diaph3 and Diaph3-M1041A interacted with the E3 ubiquitin ligase Stub1. *D* and *E*, Western blots (*D*) and quantitative data (*E*) showed that Stub1 overexpression promoted the degradation of Diaph3-M1041A with or without CHX treatment. Mean ± SD, ∗*p* < 0.05, ∗∗*p* < 0.01 by unpaired Student’s *t* test. *F*, Stub1 overexpression decreased SRF activity of Diaph3-M1041A. Mean ± SEM. ∗∗*p* < 0.01 by unpaired Student’s *t* test. *G*, Stub1 knockdown increased Diaph3-M1041A expression. *H* and *I*, Protein quantification of Stub1 (*H*) and Diaph3-M1041A (*I*) in panel *G*. Mean ± SD, ∗*p* < 0.05, ∗∗*p* < 0.01 by unpaired Student’s *t* test. *J*, Stub1 knockdown did not affect SRF activity of Diaph3-M1041A. Mean ± SEM. ns, not significant. *K*, The E1 inhibitor TAK-243 did not further increase Diaph3-M1041A protein levels after Stub1 knockdown. Cells were treated with 5 μM TAK-243 for 6 h. *L*, Protein quantification of Stub1 and Diaph3-M1041A in panel *K*. Mean ± SD. ns, not significant, ∗*p* < 0.05, ∗∗∗*p* < 0.001 by unpaired Student’s *t* test. CHX, cycloheximide; SRF, serum response factor.

In order to identify the E3 ubiquitin ligase mediating the degradation of Diaph3 after disruption of autoinhibition (or M1041A mutation), proteins associated with full-length WT Diaph3-Flag or Diaph3-M1041A-Flag overexpressed in HEK293T cells were coimmunoprecipitated by Flag antibody and subjected to mass spectrometry (Fig. 3*A*). A total of five E3 ubiquitin ligases were identified to interact with Diaph3-M1041A, including Stub1, Fbxo3, Anapc4, Trim27, and Skp1 (Fig. 3*B*, Table S1). Co-immunoprecipitation (Co-IP) experiments validated that Stub1, Fbxo3, Anapc4, and Trim27, but not Skp1, interacted directly with WT and M1041A Diaph3 (Fig. 3*C*, S3, *A*–*D*). Importantly, Diaph3-M1041A protein level was significantly reduced after coexpression of Stub1, while the other E3 ubiquitin ligases had little effects (Fig. 3, *D* and *E*, S3, *E*–*L*). Consistently, overexpression of Stub1 also decreased the actin polymerization activity of Diaph3-M1041A (Fig. 3*F*). To further validate the regulation of Diaph3-M1041A by Stub1, we cotransfected the Stub1-shRNA with Diaph3-M1041A into HEK293T cells. Efficient knockdown of the endogenous Stub1 was validated by Western blot analysis (Fig. 3, *G* and *H*). Importantly, Stub1 knockdown significantly increased the expression of Diaph3-M1041A protein (Fig. 3, *G* and *I*). No effect was observed on the actin polymerization activity of Diaph3-M1041A after Stub1 knockdown (Fig. 3*J*), likely due to saturation of the Diaph3-M1041A activity. To explore if other E3 ligases may also contribute to degradation of Diaph3, E1 ligase inhibitor TAK-243 was treated alongside the Stub1 depletion. TAK-243 treatment promoted stabilization of Diaph3-M1041A in the presence of Stub1; however, TAK-243 did not further increase the expression of Diaph3-M1041A after Stub1 knockdown (Fig. 3, *K* and *L*).These results suggested that Stub1 is the primary E3 ubiquitin ligase that mediates the degradation of the active Diaph3-M1041A protein.

### Stub1 regulates the activity of WT Diaph3

**Figure 4:**
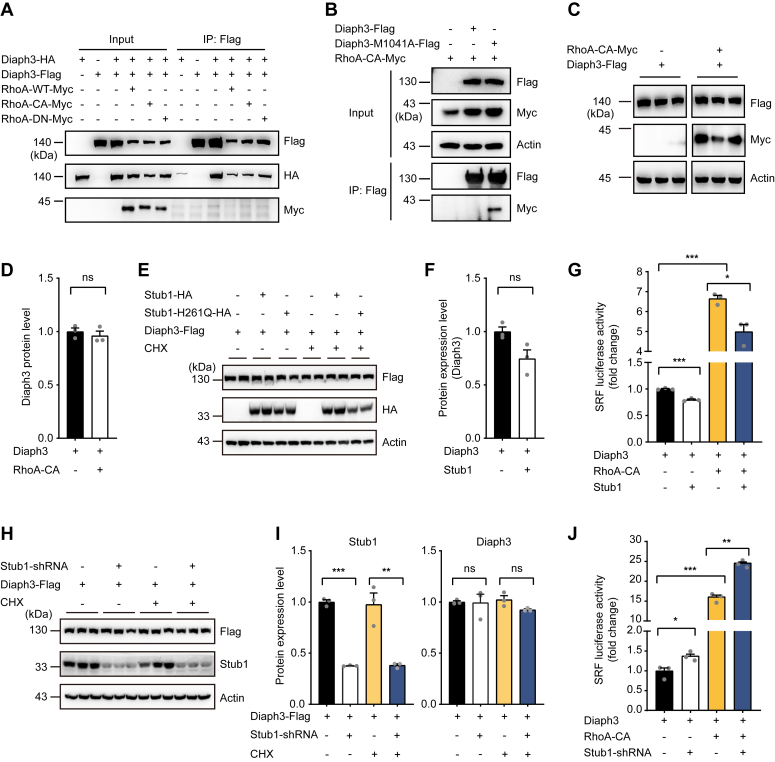
**Stub1 modulates the activity of WT Diaph3.***A*, RhoA did not interact with full length Diaph3. *B*, Diaph3-M1041A but not full length Diaph3 interacted with RhoA-CA. *C*, RhoA did not affect protein stability of the full length Diaph3. *D*, protein quantification of Diaph3-Flag in panel *C*. Mean ± SD. ns, not significant. *E*, Stub1 overexpression did not affect the expression of WT Diaph3 with or without CHX treatment. *F*, protein quantification of Diaph3-Flag after Stub1 overexpression. Mean ± SD. ns, not significant. *G*, Stub1 overexpression decreased SRF activity of the WT Diaph3 in the presence or absence of RhoA-CA. Mean ± SEM. ∗*p* < 0.05, ∗∗∗*p* < 0.001 by unpaired Student’s *t* test. *H*, Stub1 knockdown did not affect the expression of WT Diaph3 with or without CHX treatment. *I*, protein quantification of Stub1 and Diaph3-Flag in panel *H*. Mean ± SD. ns, not significant, ∗∗*p* < 0.01, ∗∗∗*p* < 0.001 by unpaired Student’s *t* test. *J*, Stub1 knockdown increased SRF activity of the WT Diaph3 in the presence or absence of RhoA-CA. Mean ± SEM. ∗*p* < 0.05, ∗∗*p* < 0.01, ∗∗∗*p* < 0.001 by unpaired Student’s *t* test. SRF, serum response factor.

We next explored if Stub1 also regulates the expression and activity of the activated WT Diaph3. Physiologically, Diaph3 protein is autoinhibited by its DID-DAD interaction and activated by binding to the active Rho GTPase. Although the N-terminal fragment of Diaph3 interacted with RhoA-CA (Fig. 2*E*), we failed to detect interaction between the full-length Diaph3 and RhoA constructs, including RhoA-CA (Fig. 4*A*). This is consistent with previous report that Diaph3 exhibits strong autoinhibitory interaction between its DID and DAD domains, and the interruption of this interaction *in vitro* would require the addition of a 20-fold molar excess of active RhoA (26). As M1041A mutation abolished the autoinhibition by DID-DAD interaction, full-length Diaph3-M1041A resumed its interaction with RhoA-CA (Fig. 4*B*). RhoA-CA did not affect the expression of the full-length Diaph3 (Fig. 4, *C* and *D*), consistent with the lack of detectable interaction between RhoA-CA and full-length Diaph3.

Interestingly, although Stub1 overexpression failed to reduce the expression of WT Diaph3 (Fig. 4, *E* and *F*), it significantly reduced the actin polymerization activity of Diaph3, both in the absence and presence of RhoA-CA (Fig. 4*G*). Of particular note, RhoA-CA induced the activity of WT Diaph3 (Fig. 4*G*) albeit lack of detectable interaction between the two molecules (Fig. 4*A*), suggesting that a very small proportion of Diaph3 may be bound to and activated by RhoA-CA while the majority of the Diaph3 proteins remain autoinhibited. Similarly, although Stub1 knockdown failed to increase the expression level of the WT Diaph3 (Fig. 4, *H* and *I*), it significantly increased the actin polymerization activity of Diaph3, both in the absence and presence of RhoA-CA (Fig. 4*J*). Thus, our results indicated that RhoA-CA binds to and activates a small proportion of the WT Diaph3 (below detection limit), which are subjected to modulation by the E3 ubiquitin ligase Stub1.

### Stub1 mediates polyubiquitination of Diaph3-M1041A at K1142-K1143-K1144 residues

**Figure 5:**
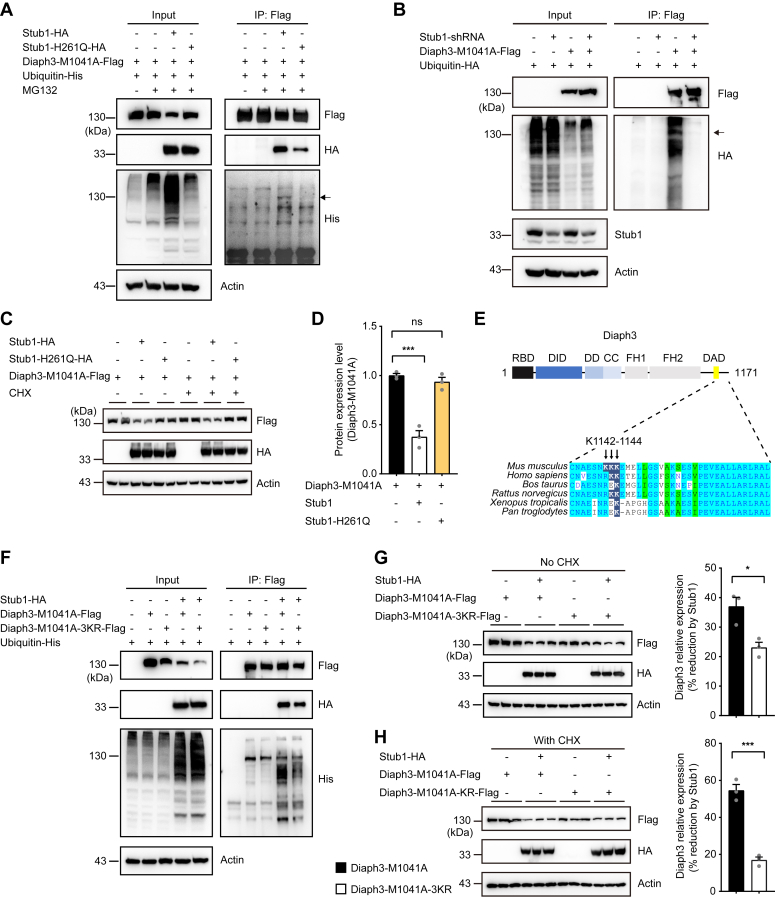
**Stub1 mediates polyubiquitination of Diaph3-M1041A.***A*, overexpression WT Stub1 but not Stub1-H261Q mutant induced Diaph3-M1041A polyubiquitination. The *arrow* indicates ubiquitin covalently bound to the lysine residues of Diaph3-M1041A (140 kD). *B*, Stub1 knockdown reduced Diaph3-M1041A polyubiquitination. *C*, overexpression of WT Stub1 but not Stub1-H261Q mutant reduced Diaph3-M1041A protein expression with or without CHX treatment. *D*, protein quantification of Diaph3-M1041A-Flag after Stub1 or Stub1-H261Q overexpression. *E*, conservation of the Diaph3 K1142 to 1144 residues in different mammalian species. *F*, the 3 KR mutation reduced Diaph3-M1041A polyubiquitination by Stub1. *G* and *H*, Western blots and quantitative data showed that the 3KR mutation partially counteracted the decrease in Diaph3-M1041A expression caused by Stub1 overexpression without CHX (*G*) or with CHX (*H*). Mean ± SD. ns, not significant, ∗*p* < 0.05, ∗∗∗*p* < 0.001 by unpaired Student’s *t* test.

To further verify if Stub1 regulates the expression of Diaph3-M1041A *via* ubiquitination, we examined the effect of WT and catalytically inactive (H261Q) Stub1 on ubiquitination levels of Diaph3-M1041A. Overexpression of the WT Stub1 but not Stub1-H261Q mutant results in global increase in ubiquitination levels (Fig. 5*A*), consistent with previous report (27). Correspondingly, WT but not H261Q Stub1 promoted polyubiquitination of Diaph3-M1041A (Fig. 5*A*). Furthermore, knockdown of Stub1 significantly impaired polyubiquitination of Diaph3-M1041A protein (Fig. 5*B*). The catalytically inactive Stub1-H261Q also failed to reduce the expression of Diaph3-M1041A (Fig. 5, *C* and *D*), consistent with its inability to polyubiquitinate the Diaph3-M1041A protein (Fig. 5*A*).

We next used the BDM-PUB algorithm (http://bdmpub.biocuckoo.org/prediction.php) (28) to predict the candidate lysine residues on mouse Diaph3-M1041A protein that may be ubiquitinated by Stub1. Lysine residues at K1142, K1143, and K1144 on Diaph3 were predicted to be potential ubiquitination sites. We compared the conservation of Diaph3 K1142 to 1144 residues in different mammals. K1143 to 1144 residues are conserved in human (Fig. 5*E*). Indeed, mutations of the three lysine residues to arginine (Diaph3-M1041A-3KR) partially abolished polyubiquitination of Diaph3-M1041A by Stub1 (Fig. 5*F*). Similarly, Diaph3-M1041A-3KR was more resistant to Stub1-mediated protein degradation, compared to the Diaph3-M1041A control (Fig. 5, *G* and *H*). As Diaph3-M1041A-3KR only partially rescued the expression level of Diaph3-M1041A (Fig. 5, *F–H*), other lysine residues (including those located at the N terminus) may also contribute to Diaph3 polyubiquitination and degradation. Together, these results suggested that the E3 ligase Stub1 mediates polyubiquitination of the activated Diaph3, partially at K1142 to 1144 residues.

### Knockdown of the endogenous Stub1 in mouse inner ear leads to severe hearing loss

**Figure 6:**
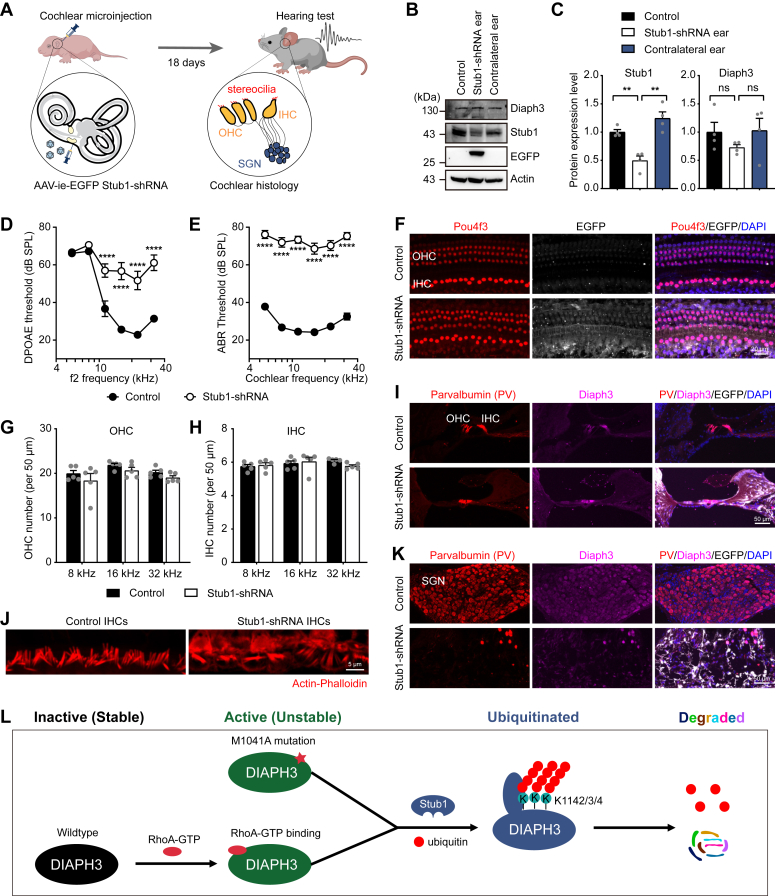
**Knockdown of the endogenous Stub1 in mouse inner ear leads to severe hearing loss.***A*, schematic diagram of knocking down endogenous Stub1 expression in mouse inner ear. AAV-ie vector containing GFP tag was used to package Stub1-shRNA. The left ear of P3 mice were injected with AAV-ie *via* round window membrane (RWM). Hearing measurements was performed 18 days (P21) after injection. Cochlear histology was performed to examine survival and morphology of hair cells, stereocilia bundles and spiral ganglion neurons (SGNs). IHC, inner hair cell; OHC, outer hair cell. *B* and *C*, Western blot images (*B*) and protein quantification (*C*) of Stub1 and Diaph3 expression in control and Stub1 knockdown cochlea. Mean ± SD. ns, not significant, ∗∗*p* < 0.01 by unpaired Student’s *t* test. *D* and *E,* DPOAE (*D*) and ABR (*E*) thresholds in control and Stub1 knockdown mice at P21. n = 18 ears each condition. Mean ± SEM. ∗∗∗∗*p* < 0.0001 by two-way ANOVA followed by Bonferroni's post test. SPL, sound pressure level. ABR, auditory brainstem response; DPOAE, distortion product otoacoustic emission. *F*, wholemount immunofluorescent images of hair cells labeled with anti-Pou4f3 antibody (*red*) in control and Stub1 knockdown cochlea at P21. *G* and *H*, numbers of outer hair cells (*G*) and inner hair cells (*H*) at different cochlear frequencies (8, 16, 32 kHz) (n = 5 cochleae each condition). Mean ± SEM. *I*, immunofluorescent images showing Parvalbumin (hair cells) and Diaph3 expression in cryo-sectioned cochlea (organ of Corti area). *J*, hair cell stereocilia bundle morphology labeled with Actin-Phalloidin. *K*, immunofluorescent images showing Parvalbumin (SGNs) and Diaph3 expressions in cryo-sectioned cochlea (SGN area). *L*, proposed mechanism of a novel negative feedback regulation of Diaph3 protein expression and activity by Stub1-mediated degradation. AAV-ie, AAV-inner ear.

Overexpression of Diaph3 is associated with auditory neuropathy in human patients and mouse models (8, 9, 10, 29). To explore the *in vivo* functions of Stub1 in the auditory system, we used AAV-inner ear (AAV-ie) virus to knockdown Stub1 in cochlear cells. AAV-ie can transduce hair cells, supporting cells, and spiral ganglion neurons (SGNs) when injected through round window membrane (RWM) of the mouse cochlea (30). The AAV-ie vector contains the mouse Stub1 shRNA driven by the U6 promoter and the EGFP protein driven by the cytomegalovirus promoter. These AAV-ie vectors were injected into C57BL/6 mice through the RWM at postnatal day 3 (P3), and auditory function tests and histological analyses were conducted at postnatal day 21 (P21) (Fig. 6*A*).

Efficient knockdown of the Stub1 protein in the cochlea was validated by Western blot analyses (Fig. 6, *B* and *C*). Stub1 knockdown did not significantly affect the total protein level of Diaph3 (Fig. 6, *B* and *C*), consistent with the results from cultured cells (Fig. 5, *H* and *I*). Auditory functions of the injected mice were evaluated by the distortion product otoacoustic emission (DPOAE) and auditory brainstem response (ABR) tests. Elevated DPOAE and ABR thresholds indicate impaired outer hair cell function and neural transmissions, respectively. Mice injected with AAV-ie-EGFP Stub1 shRNA exhibited elevated DPOAE thresholds (Fig. 6*D*) and ABR thresholds (Fig. 6*E*) compared with the control, indicating that Stub1 knockdown resulted in significant impairment in auditory functions. To understand the pathological consequences of Stub1 knockdown, cochlear hair cells, stereocilia bundles, and SGNs were examined. Interestingly, despite profound hearing loss, numbers of hair cells were unaffected after Stub1 knockdown in cochlear wholemount samples (Fig. 6, *F*–*H*). Similarly, cryo-sections also displayed normal morphologies of the organ of Corti and hair cells (Fig. 6*I*). Intriguingly, the actin-rich stereocilia bundles of the hair cells were highly disorganized with fusion and elongations after Stub1 knockdown (Fig. 6*J*). This is consistent with hair cell stereocilia phenotype observed in mice overexpressing Diaph3 (10, 29). Furthermore, Stub1 knockdown also lead to significant degeneration of the SGNs (Fig. 6*K*), highlighting an essential role of Stub1 in promoting SGN survival. Similarly, mice overexpressing Diaph3 also showed loss of synapses, which connect hair cells and SGNs (29). In summary, these findings indicate that knocking down endogenous Stub1 expression in the mouse cochleae results in the abnormal morphology of the hair cell stereocilia, degeneration of SGNs and severe hearing loss, resembling the phenotypes observed in mice overexpressing Diaph3.

## Discussion

Study on the Diaph3 transgenic mice revealed that the mouse overexpressing Diaph3 mimicked the human auditory neuropathy phenotype (10). However, KO of the Diaph3 gene in mice resulted in developmental defect but not hearing loss (31). Distinct pathological features derived from Diaph3 overexpression (hyperactivity) and deletion (hypoactivity) highlight the importance of precise control of Diaph3 expression and activation in cellular functions. In this study, we identify a novel mechanism for negative feedback regulation of Diaph3 protein expression and activity (Fig. 6*L*). Under the basal condition, majority of the Diaph3 proteins are present as autoinhibited, inactive, and stable forms. When a small amount of Diaph3 proteins are activated by RhoA GTPase, the intramolecular DID-DAD interaction is disrupted (similar to the M1041A mutation), allowing actin polymerization by FH1/FH2 domains. Meanwhile, ubiquitination sites, including K1142 to 1144 within the DAD domain, of the activated Diaph3 protein become accessible and are ubiquitinated by the E3 ligase Stub1, leading to degradation of the active Diaph3 (Fig. 6*L*). The selective degradation of the activated Diaph3 by Stub1 contributes to the fine-tuned control of actin polymerization mediated by Diaph3.

Actin polymerization activities of the diverse formin family members are modulated by distinct mechanisms owning to their diverse domain structures. Daam1 is activated in a different manner than Diaph3, in which the PDZ domain of the Disheveled (Dvl) protein binds to the DAD of Daam1, thereby releasing autoinhibition (32). Actin binds to the WH2 domain of the formin INF2, which therefore competes for the intramolecular FH3–WH2 domain autoregulatory interaction and abolishes the autoinhibition. Such mechanism would be self-regulated by the concentration of actin molecules, which act as both antagonists for WH2 binding and as substrates for actin filament elongation (33). Delphilin contains two N-terminal PDZ domains that typically interact with short signature motifs occurring in flexible regions of their target proteins, which is again reminiscent to the GTPase binding domain/FH3–DAD autoregulation interaction (34, 35). Therefore, selective degradation of the activated Diaph3 serves as a new dimension for formin regulation. Whether similar mechanism is applicable to other DRFs or formin family members remains to be determined.

Additionally, Diaph3 and its family DRF proteins are regulated by diverse array of binding partners, including Rho GTPases, EB1, adenomatous polyposis coli protein, IRSp53, Bud6p/Aip3, and anillin and so on (36, 37, 38). RhoA-dependent activation of Diaph3 is enhanced by binding to anillin to facilitate local assembly of β-actin filaments at the cytokinetic furrow (38). The stabilizing activity of DRF proteins on microtubules can be achieved by their interaction with other microtubule-interacting proteins such as EB1 and adenomatous polyposis coli (39). In this study, we identify Stub1 as the candidate E3 ligase for degradation of the activated Diaph3. However, the fate of activated Diaph3 may be highly dependent on its conformation and binding partners, whereby it can be stabilized for continuous cytoskeletal remodeling or degraded to prevent excessive actin polymerization. Recently, Diaph3 was found to undergo liquid-liquid phase separation that acts as a regulatory hub for stress-induced actin cytoskeleton remodeling (40). Phase separated Diaph3 accumulates in Diaph3 granules (D-granules) to inhibit assembly of actin filaments in filopodia. Interestingly, the liquid-liquid phase separation is regulated by low complexity region 2 containing DD, FH1, and part of FH2 domains. Whether inactive (autoinhibited) or active Diaph3 proteins may condensate at similarly propensity remains unknown.

Stub1-mediated ubiquitination and degradation of the active Diaph3 represents a negative feedback regulatory mechanism to restrain Diaph3 activity. Such regulatory mechanism by selective degradation of the activated proteins is not uncommon. Circadian photoresponses are regulated by light-activated cryptochrome (CRY) in *Drosophila melanogaster*. CRY is rapidly degraded in its active form in light and accumulates in darkness. CRY lacking its C terminus exhibits light-independent constitutive activity similar to the WT CRY under continuous light, suggesting that the C terminus of CRY regulates the activity and degradation of the photosensitive, photolyase-like portion of the protein (41). Phosphatase and tensin homolog deleted on chromosome 10 (PTEN) is a potent tumor suppressor and a multifunctional signaling protein under tight regulation. The catalytic N-terminal C2 phosphatase domain acts on protein and lipid substrates, while its C terminus serves as an autoinhibitory domain to control PTEN membrane recruitment and phosphatase activity (42, 43). PTEN is a relatively stable protein, truncation of its C terminus leads to rapid degradation of PTEN mediated by Nedd4-1. This suggests that the intramolecular interactions between the C-terminal and N-terminal C2 domains of PTEN play a key role in stabilizing PTEN by inhibiting the PTEN-NEDD4-1 interactions (44, 45).

Stub1 is an essential E3 ligase involved in protein quality control. Recent studies have shown that Stub1 is involved in regulating various pathophysiological functions, including autophagy and lysosomal functions (46, 47), aging (48), male reproduction (49, 50), bone remodeling (51), neurodegeneration (52, 53, 54), and cardiovascular disorders (55). In this study, we found that Stub1 deficiency in mouse inner ear leads to hair cell stereocilia defects, SGN degeneration, and hearing loss. These phenotypes bear remarkable resemblance to the mouse model overexpressing transgenic Diaph3 (10, 29). Therefore, agonists or inhibitors of Stub1 may serve as potential therapeutic treatments for various disorders, particularly autosomal dominant nonsyndromic auditory neuropathy caused by overexpression of Diaph3.

## Experimental procedures

### Materials

The HEK293T cell line was purchased from the Chinese Academy of Sciences Cell Culture Collection. Antibodies were from the following companies: anti-Flag (SM009100) and anti-HA (SA068005) magnetic beads from Changzhou Smart-Lifesciences Biotechnology, China; Flag (14793S) and HA (3724S) antibodies from Cell Signaling Technology; Actin (A1978) antibody from Sigma-Aldrich; Myc, His (30401ES10) and EGFP (31002ES60) antibodies from Yeasen, China; Stub1 (55430-1-AP) antibody from Proteintech; Diaph3 (DP3491) antibody from ECM Biosciences. Specificities of the primary antibodies were validated in cells transfected with corresponding plasmids. Horseradish peroxidase-conjugated secondary antibodies including anti-rabbit immunoglobulin G (BS13278) and anti-mouse IgG (BS12478) were from Bioworld, China. CHX (66-81-9) was from Sigma-Aldrich and MG132 (1211877-36-9) was from Selleck Chemicals. TAK-243 (HY-100483) was from MedChemExpress.

### Animal

The use of animals was following the standard ethical guidelines. The C57BL/6 mice used in the experiment (GemPharmatech Inc) had an equal sex ratio. Animals were housed at a room temperature of 22 ± 1 °C, humidity of 55% ± 5%, light/dark cycle for 12 h, and plenty of available food and water. All animal experiments were approved by the Institutional Animal Care and Use Committee of Model Animal Research Center of Nanjing University, China (#WGQ04).

### Plasmid construction

Diaph3 (NM_019670.2), Stub1 (NM_019719.4), Fbxo3 (NM_012175), Anapc4 (NM_024213.2), Trim27 (NM_009054.3), Skp1 (NM_011543.4), and RhoA (NM_001313941.2) Complementary DNA sequences were cloned to the eukaryotic expression vector pcDNA3.1. Mutated and truncated fragments were derived from the above-mentioned plasmids. All mammalian expression plasmids were amplified in DH5α bacteria, and all plasmids were validated by sequencing and transfection experiments.

### Cell culture

HEK293T cells were cultured in Dulbecco's modified Eagle's medium supplemented with 10% fetal bovine serum, glutamine, nonessential amino acids, sodium pyruvate, and penicillin-streptomycin. Plasmids were transfected into the cells using liposomal transfection reagents according to the manufacturer's instructions. HEK293T cells were cultured in a humidified 37 °C incubator containing 5% CO_2_.

### Coimmunoprecipitation

After transfection, cells were harvested 48 h later, appropriate lysis buffer (including protease inhibitors) was added, supernatant was centrifuged after full lysis, and a small amount of lysate was taken for Western blot analysis. The remaining lysate was incubated with Flag beads at 4 °C overnight. After immunoprecipitation, the supernatant was discarded and the beads washed with 1 ml lysis buffer for 3 to 4 times. Finally, 30 μl of 2×SDS loading buffer was added and boiled in a metal bath at 95 ˚C for 10 min. Protein samples were obtained after centrifugation. Samples were subjected to the Western blot analysis.

### Stub1 shRNA knockdown

For Stub1 knockdown, target sequences for silencing human Stub1 were cloned into pLKO1, a lentiviral vector with puromycin resistance screening label and GFP tag. Plasmids of Stub1 shRNA were constructed with forward (5′- CCG GGA AGA GGA AGA AGC GAG ACA TCT CGA GAT GTC TCG CTT CTT CCT CTT CTT TTT G -3′) and reverse (5′- AAT TCA AAA AGA AGA GGA AGA AGC GAG ACA TCT CGA GAT GTC TCG CTT CTT CCT CTT C -3′) primers. Sticky ends for subcloning were generated by restriction enzymes *EcoRI* and *AgeI*. The knockdown efficiency of Stub1 in HEK293T cells was examined by Western blot.

### PAGE and Western blotting

After transfection, cells were harvested at either 24 h later (unless otherwise indicated in figure legends), lysis buffer (50 mM Tris–HCl pH 7.4, 150 mM NaCl, 1 mM EDTA pH 8.0, 1% Triton X-100, and 1 mM phenylmethylsulfonyl fluoride, protease inhibitor) was added, and lysis was performed at 4 ˚C for 10 min. The protein samples were then prepared by homogenization or centrifugation. All protein samples were subjected to SDS-PAGE and transferred to polyvinylidene fluoride membrane, followed by antibody incubations. The nondenaturing PAGE was performed similarly without adding denaturing agents including SDS and mercaptoethanol. Electrophoresis was performed at 0 to 4 ˚C to prevent overheating. Signals from horseradish peroxidase-conjugated secondary antibodies were visualized using the enhanced chemiluminescence substrate (180-5001, Tanon) on the automatic chemiluminescence image analysis system (4600, Tanon).

### Mass spectrometry proteomic analysis

After transfection of plasmid for 48 h, MG132 was treated for 6 h, and cell lysate was collected for immunoprecipitation using Flag antibodies. The Co-IP enriched protein complex were shortly separated by SDS-PAGE and subjected to coomassie blue G250 staining. The subsequent mass spectrometry proteomics analysis was carried out as previously described (56). Briefly, the gel bands containing proteins of interest were excised and digested by trypsin. The resulting peptides were extracted and finally submitted to liquid chromatography with tandem mass spectrometry analysis using a nanoLC.2D (Eksigent Technologies) coupled with a TripleTOF 5600 System (SCIEX). The original mass spectrometry data were submitted to ProteinPilot Software (https://sciex.com/products/software/proteinpilot-software) (version 4.5, SCIEX) for database searching against UniProt Homo Sapiens database concatenated with reverse decoy database. All the searching parameters in ProteinPilot were set to default values. In addition, Protein Prospector (https://prospector.ucsf.edu) (version 5.20.0) was used for search compare and protein quantification as described previously.

### SRF-RE dual-luciferase reporter assays

The pGL4.34[luc2P/SRF-RE/Hygro] plasmid was kindly provided by Dr Cheng Deng (Sichuan University, China). A pRL-TK renilla luciferase reporter plasmid was used as an internal reference for normalizing the firefly luciferase reporter. Various Diaph3 constructs were cotransfected with pGL4.34 and pRL-TK plasmids, and starved for 6 h prior to the harvest. The cell lysates were collected and the SRF activity, which indirectly represents Diaph3 actin polymerization activity, was assessed using a dual luciferase assay kit (RG089M, Beyotime).

### Immunofluorescence and imaging

For cochlear histological analysis, the mouse cochlea was isolated after audiometry. Isolated mouse cochleae were fixed in 4% paraformaldehyde for 2 h with shaking at room temperature, and then decalcified in 5% EDTA for 3 days. Decalcified cochleae were used for microdissection and cryosection. After microdissection and cryosection, dissected cochlear samples were blocked in 5% heat inactivated horse serum with 0.3% Triton X-100 in PBS for 1 h, and then incubated with primary antibodies overnight at 4 °C. Next day, samples were rinsed in PBS for 3 times and incubated with secondary antibodies for 2 h at room temperature. Primary antibodies used in this study, as follows: rabbit anti-EGFP (1:400, 31002ES60, Yeasen), mouse anti-Pou4f3 (1:200, sc-81980, Santa Cruz), mouse anti-Parvalbumin (1:500, sab4200545, Sigma-Aldrich), rabbit anti-Diaph3 (1:400, DP3491, extracellular matrix). Secondary antibodies used in this study are as follows: Alexa Fluor 488 AffiniPure Goat Anti-Rabbit IgG (H + L) (1:500, 111-545-003, Jackson ImmunoResearch), Alexa Fluor 568-conjugated goat anti-mouse (IgG1) (1:500; A21124, Life Technologies), Alexa Fluor 647 AffiniPure Goat Anti-Rabbit IgG (H + L) (1:500, 111-605-003, Jackson ImmunoResearch). Nuclei were stained with 4′,6-diamidino-2-phenylindole (1:2000, 28718-90-3, Roche). F-actin was stained with Phalloidin-iFluor 594 (1:1000, ab176757, Abcam).

Samples were analyzed using the inverted fluorescence microscope (XD, SOPTOP), Leica SP5 confocal microscope (Leica TCS SP5, Leica Microsystems). ImageJ software (https://imagej.net/ij) was used to process image, map cochlear length to cochlear best frequencies.

### Mouse Stub1 shRNA AAV-ie virus preparation

For Stub1 knockdown *in vivo*, target sequences for silencing mouse Stub1 were cloned into the AAV plasmid containing the cytomegalovirus promoter, GFP tag. and polyA tail. Plasmids of Stub1 shRNA were constructed with forward (5′- CCG GCC CTT CGC ATT GCT AAG AAG ACT CGA GTC TTC TTA GCA ATG CGA AGG GTT TTT G -3′) and reverse (5′- AAT TCA AAA ACC CTT CGC ATT GCT AAG AAG ACT CGA GTC TTC TTA GCA ATG CGA AGG G -3′) primers. Sticky ends for subcloning were generated by restriction enzymes EcoRI and AgeI. mStub1-shRNA (AAV2/i.e., 1 × 10^13^ genomic copies per mL) were made by PackGene Biotech. The knockdown efficiency of Stub1 in mouse cochlear was examined by Western blot.

### RWM injection

To infect the cochlear tissue with AAV-ie virus, P3 mice were selected for injection. Low temperature anesthesia was used to minimize the damage of anesthesia to mice. P3 mice were placed in an ice bath for 2 to 3 min until loss of consciousness and then moved to the ice pad for the follow-up surgery. Surgery time was limited to 5 to 10 min. The left ear of each pup was injected, and the noninjected contralateral right ear was used as a negative control. After anesthesia, the otic bulla was exposed by postauricular incision to visualize the cochlea, RWM was exposed by examining the relative position of the facial nerve to the temporal bone. Glass micropipette was used to inject about 2 μl AAV per cochlea. After injection, tissue adhesives (1469SB, 3M Vetbond) were used to bind the skin incisions, and the pups were placed under a heat lamp. The awaken pups were returned to their breeding cages.

### Auditory function tests *via* DPOAE and ABR measurements

Animals were intraperitoneal injected of 375 mg/kg Avertin (Sigma-Aldrich) for anesthesia. DPOAE and ABR tests were performed in a sound-proof chamber on P21 mice as published previously (57). For ABR measurements, three needle electrodes were used, “A” placed on the dorsal midline between the two ear flaps, “B” placed behind the pinna of test ear, and “G” placed at the base of the tail. The ABR responses were evoked at six frequencies (5.6, 8, 11.3, 16, 22.6, and 32 kHz). Sound pressure levels (SPLs) were raised at 5 dB-step from 10 to 80 dB (dB). The DPOAE signal in response to primary and secondary tones with frequencies f1 and f2, respectively, was recorded at the third frequency (2 × f1)-f2, with f2/f1 = 1.2, with the f2 levels 10 dB lower than the f1 levels. SPLs at the ear canal was amplified and digitally sampled at 4-ms intervals. DPOAE thresholds were defined as the f1 level required to evoke a response at −10 dB SPL. Both DPOAE and ABR recordings were performed using the EPL cochlear function test suite software (https://www.masseyeandear.org/research/otolaryngology/eaton-peabody-laboratories/engineering-core) (Mass Eye and Ear).

### Statistical analysis

Statistical tests were performed using Graphpad Prism 8 (https://www.graphpad.com). Results were reported as mean ± SEM. Specific statistical tests used in each experiment were described in figure legends. One-way ANOVA or Student’s *t* test was used for statistical analyses.

## Data availability

All data reported in this paper will be shared by the corresponding authors upon request.

## Supporting information

This article contains supporting information.

## Conflict of interest

The authors declare that they have no conflicts of interest with the contents of this article.
