# Supplementary material for: Stub1 promotes degradation of the activated Diaph3: A negative feedback regulatory mechanism of the actin nucleator
Source: J Biol Chem. 2024 Sep 23;300(10):107813. doi: 10.1016/j.jbc.2024.107813 (PMC11736009; doi:10.1016/j.jbc.2024.107813)
Supplement: Supporting Information [file mmc1.docx]

**Supporting Information**

**Stub1 promotes degradation of the activated Diaph3: a negative feedback regulatory mechanism of the actin nucleator**

Qiu et al.

**SI includes:**

1. Figures S1-3

2. Table S1

**
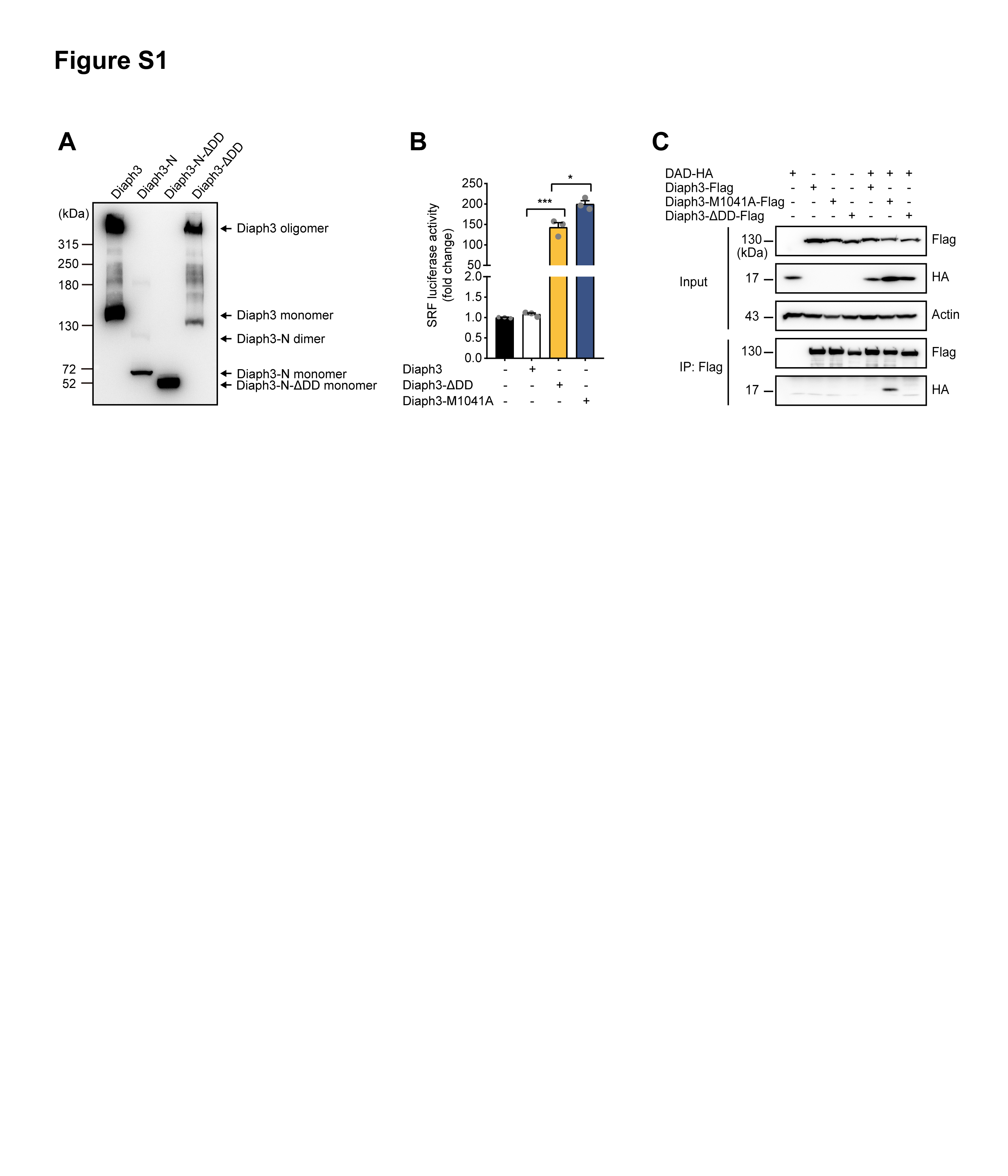
**

**Figure S1. DD-DD interaction inhibits Diaph3 activity without disrupting the DID-DAD autoinhibition.** *A*, N-terminal oligomerization of Diaph3 was mediated by intermolecular interaction of the DD-DD domains. *B*, Intermolecular interaction of the DD-DD domains inhibited Diaph3 activity. Mean ± SEM. *P < 0.05, ***P < 0.001 by unpaired Student’s t-test. *C*, Disruption of DD-DD interactions did not affect intermolecular DID-DAD autoinhibition.


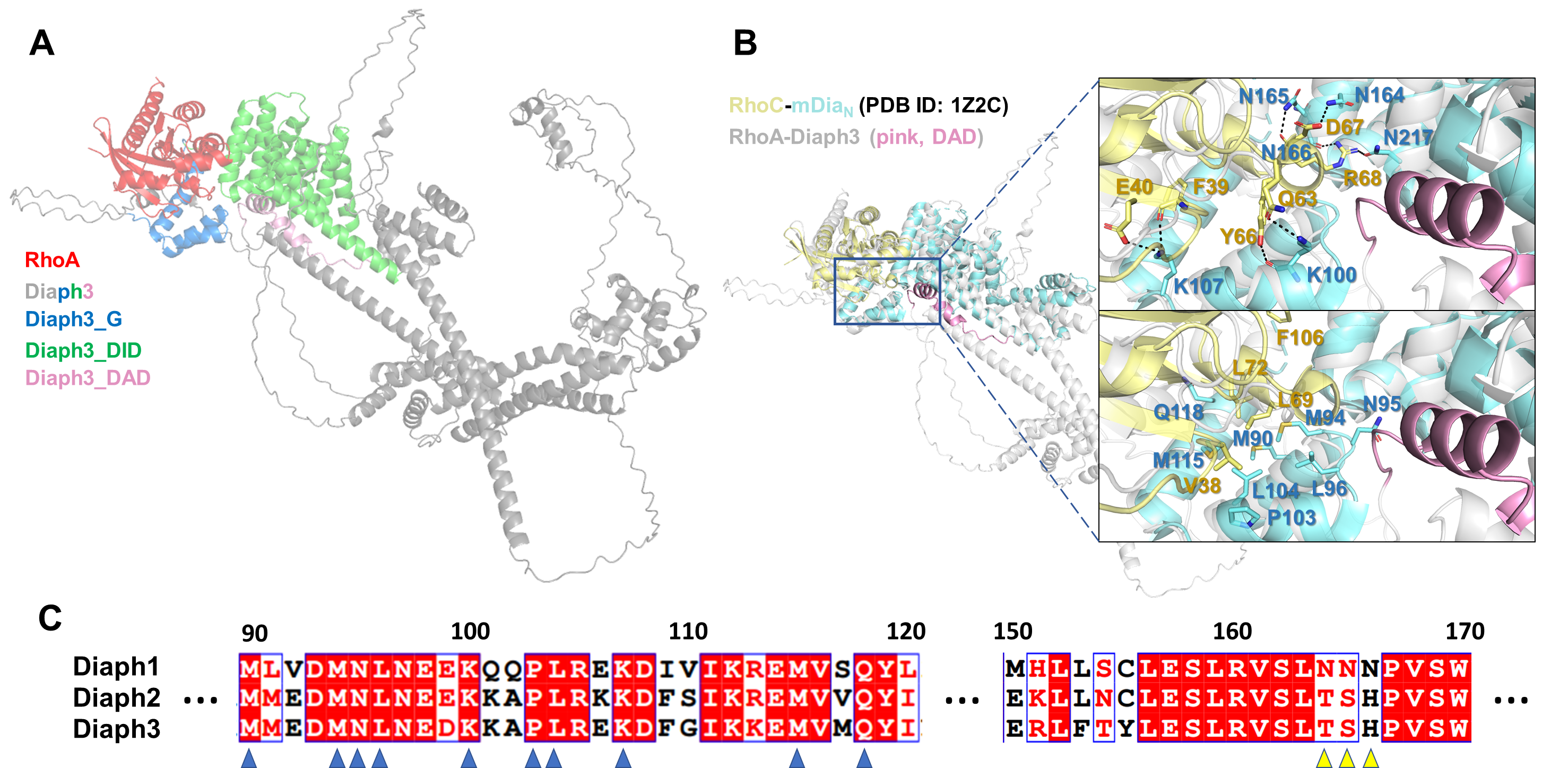


**Figure S2. Structural analysis of the RhoA-Diaph3 interaction.** *A*, Cartoon diagram of mouse RhoA-Diaph3 complex predicted by AlphaFold3. RhoA is colored by red and other part is Diaph3. G, GTPase binding region necessary for RhoA binding; DID, Diaphanous inhibitory domain; DAD, Diaphanous auto-regulatory domain. *B*, Comparison of predicted RhoA-Diaph3 complex and crystallographic structure of RhoC-mDiaN. mDiaN, N-terminal of mammal Diaph1. The detail of RhoC-mDiaN interaction is showed on the right of the image. *C*, Sequence alignment of Diaph1 (Uniprot ID: O08808), Diaph2 (Uniprot ID: O70566), and Diaph3 (Uniprot ID: Q9Z207). Key interaction residues indicated by triangles. Identical residues between Diaph1 and Diaph3 are marked with blue triangles, while differing residues are marked with yellow triangles.


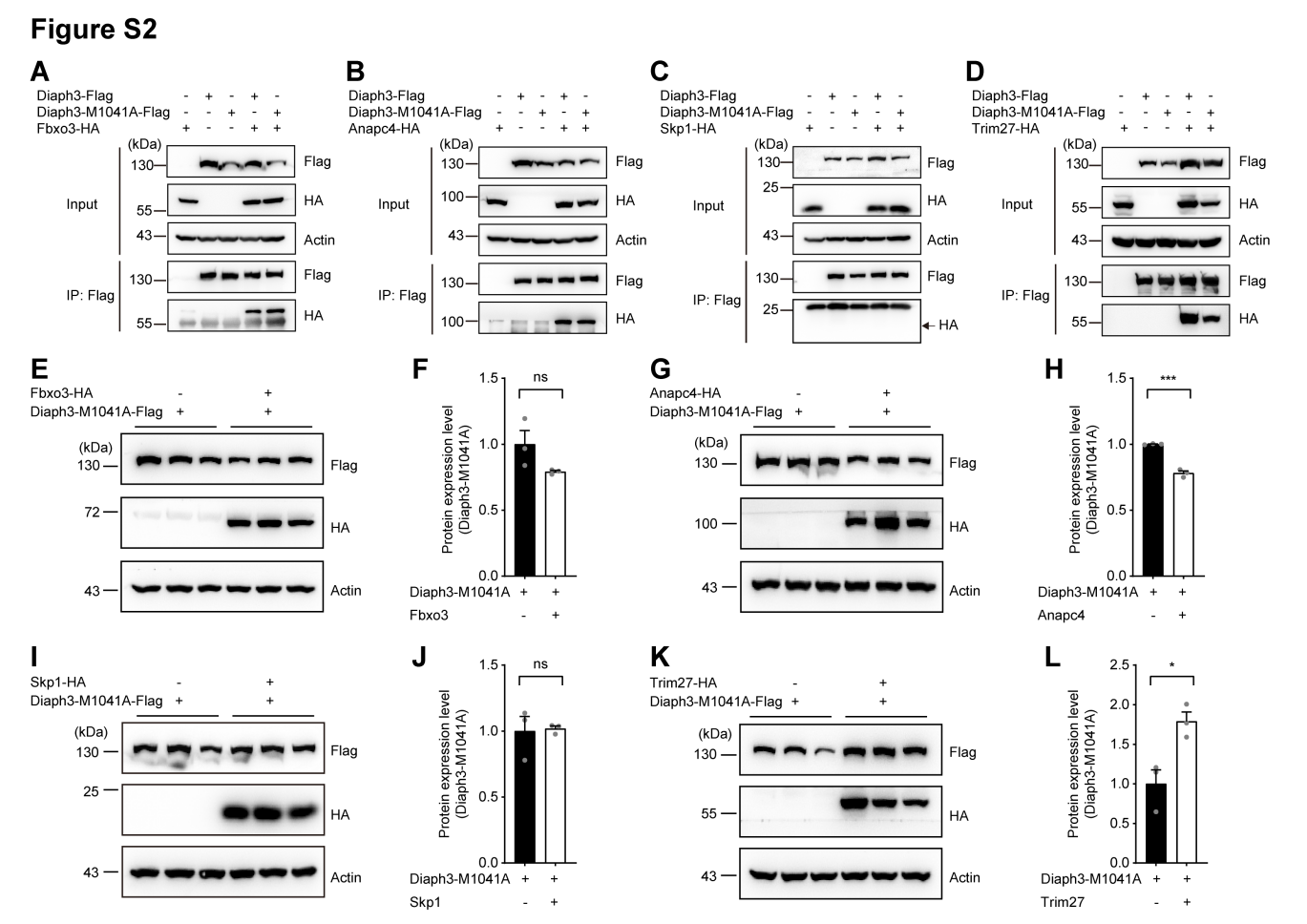


**Figure S3. Identification of candidate Diaph3 ubiquitin ligases.** *A*-*D*, WT and M1041A Diaph3 interacted with E3 ubiquitin ligases Fbxo3 (*A*), Anapc4 (*B*), Trim27 (*D*), but not Skp1 (*C*). IP with Flag. *E*, *I*, *K*, E3 ubiquitin ligases Fbxo3, Skp1 and Trim27 did not mediate the degradation of Diaph3-M1041A. *F*, *J*, *L*, Protein quantification of Diaph3-M1041A-Flag in panels *E*, *J*, *L*. *G*, E3 ubiquitin ligase Anapc4 mediated mild degradation of Diaph3-M1041A. *H*, Protein quantification of Diaph3-M1041A-Flag in panel *G*. Mean ± SD. ns, not significant, *P < 0.05, ***P < 0.001 by unpaired Student’s t-test.
